# Transverse testicular ectopia: two case reports and literature review

**DOI:** 10.1016/j.ijscr.2023.108807

**Published:** 2023-09-15

**Authors:** Ahmed Abokrecha, Ahmed Gamal Sayed, Haadi Syed, Faisal Joueidi, Lujain Alzahrani

**Affiliations:** a Department of Pediatric Surgery, Maternity and Children Hospital, Makkah, Saudi Arabia; b College of Medicine, Alfaisal University, Riyadh, Saudi Arabia

**Keywords:** Transverse testicular Ectopia, Inguinal hernia, Congenital anomaly, Case report

## Abstract

**Introduction and importance:**

Transverse Testicular Ectopia (TTE) is characterized by the presence of testis in the hemiscrotum, which can be associated with a broad spectrum of complications. It is usually manifested in pediatrics. However, on rare occasions, it can occur in adults. The diagnosis is confirmed by magnetic resonance imaging (MRI). We present two cases of Transverse Testicular Ectopia (TTE), demonstrating the significance of early diagnosis and treatment to reach optimal outcomes.

**Case presentation:**

We reported two patients with common features suggestive of Transverse Testicular Ectopia (TTE). Case 1 had open surgery; his left testis was impalpable, whereas his right side was palpable. Case 2 had undergone laparoscopy surgery, and his right and left spermatic cord was discovered on the right side.

**Clinical discussion:**

Transverse Testicular Ectopia (TTE) is classified according to clinical presentation; Type 1 is associated with inguinal hernia ranging between 40 and 50 %. Type 2 is related to persistent mullerian duct syndrome (PMDS), with a rate of 30 %. Type 3 is associated with genital anomalies and azoospermia, with a rate of 20 %. The pathogenesis is unclear. However, studies suggest that the persistence of the mullerian duct prevents normal descent of the testis. Treatment is purely dependent on early clinical presentation and surgical methods.

**Conclusion:**

Transverse Testicular Ectopia (TTE) requires delicate care by the pediatric surgeon as it is considered a rare entity in such cases. Heroin, we highlight the significance of early surgical treatment and the possibility of complications if left untreated.

## Highlights

• TTE is a rare condition, with an estimated incidence of 1 in every 4 million children.
• Imaging studies done pre-operative can be helpful to diagnosis TTE.
• The clinical presentation of transverse testicular ectopia can vary depending on the associated anomalies and age at diagnosis.

## 1 Introduction

Transverse Testicular Ectopia is a rare congenital anomaly characterized by the descent of both testes through the same inguinal canal or hemiscrotum [1,2]. Transverse Testicular Ectopia (TTE) is an even rarer occurrence, with an estimated incidence of 1 in every 4 million children [3]. Transverse Testicular Ectopia (TTE) can be associated with various complications, including inguinal hernias, testicular torsion, infertility, and testicular tumors [3]. Even though most cases are diagnosed during childhood, adult cases have also been reported [4]. The diagnosis is usually discovered incidentally during surgical exploration and by image studies [1,3]. Herion, we reported two cases of Transverse Testicular Ectopia (TTE); we aimed to share our experience and discuss the importance of early prompt diagnosis and appropriate surgical management. This case has been reported in line with the SCARE checklist [6].

## 2 Case presentation

### 2.1 First case

**Fig. 1:**
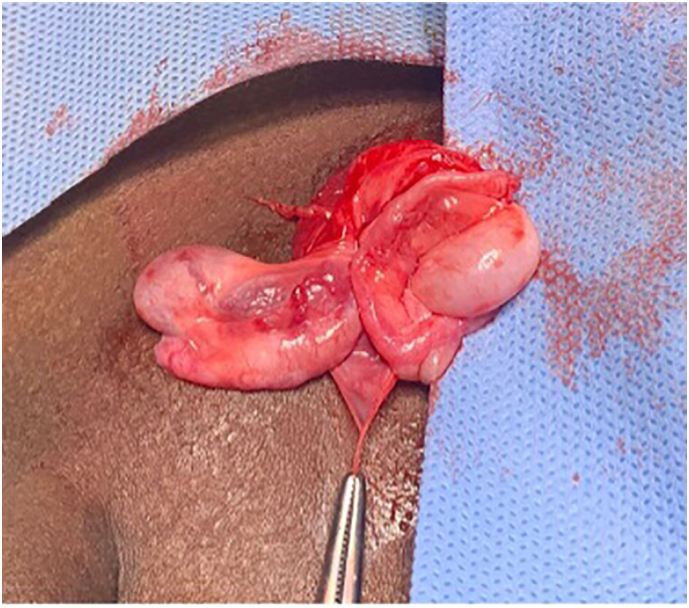
1st case. Right inguinal exploration demonstrates the right testes and spermatic cord are near the scrotum, the left testes and spermatic cord are away from the scrotum, and both testes are located in the right inguinal canal, TTE.

A 2-year-old boy from Senegal presented with bilateral undescended testes during a charity mission. The patient was born at full term and delivered vaginally without any complications. Due to lack of service, there was no attempt to orchiopexy before two years. Physical examination revealed the testes to be impalpable on the left side. The right side testis was palpable at the right inguinal area but slightly enlarged. A scrotal study revealed an empty scrotum bilaterally. Examination under general anesthesia: Due to the unavailability of a laparoscope during our charity mission, the right inguinal area was explored by open surgery. Upon opening the right inguinal region, both testes were discovered in the right inguinal canal, confirming the diagnosis of TTE (Fig. 1). A herniotomy was performed due to the presence of an associated hernia sac. The distally located one of the two testes was relocated to the contralateral side, specifically the left hemiscrotum. The proximally located testis was fixed in the right hemiscrotum. * Both testes were secured and fixed in the scrotum in a sub-dartos pouch fixation. The patient had a favorable postoperative course, and he was seen at the clinic two weeks postoperative with both testis palpable in the scrotum with suitable sizes and no reported complications during the follow-up.

### 2.2 Second case

**Figs. 2 & 3:**
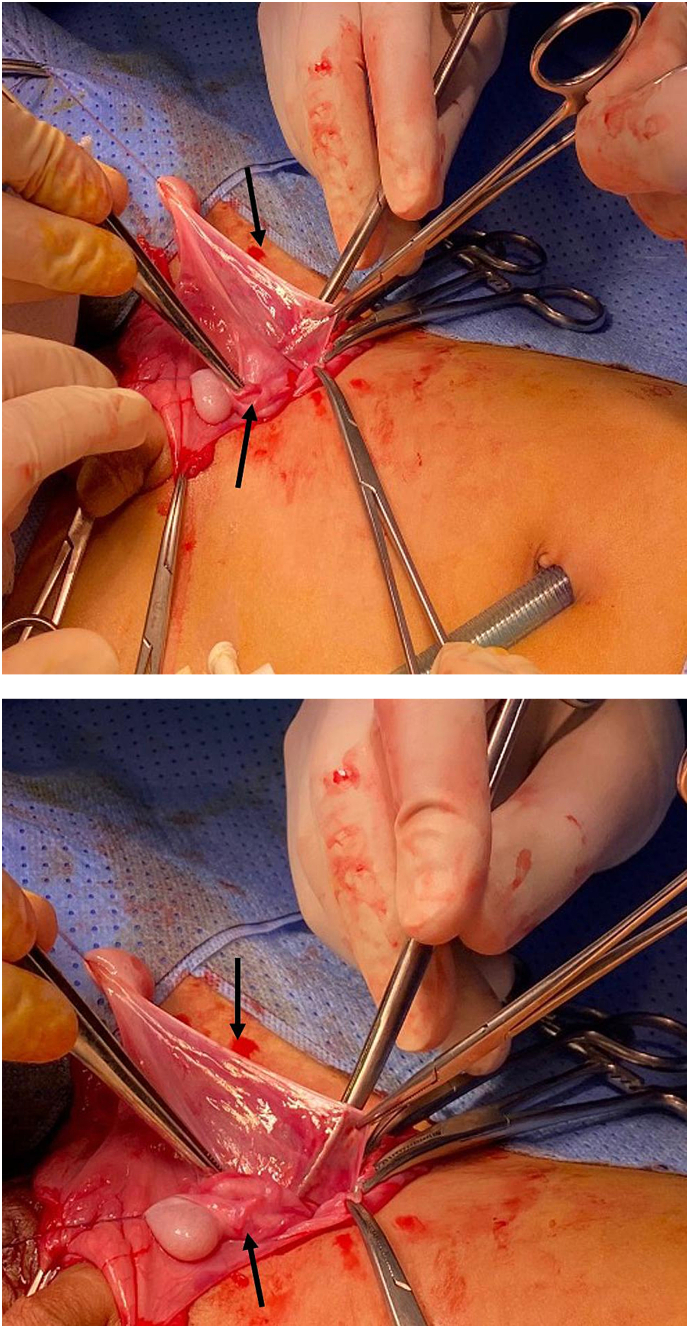
2nd case. Right inguinal exploration showed two testicules, the upper arrow show the right testes is stretched down to the scrotum with a long spermatic cord, the lower arrow show the left testes are located in the hernial sac with short spermatic cord .TTE.

A 3-year-old male who is medically free presented with left impalpable undescended testes; the right testis is in the right haemiscrotum pouch but in its upper part and right inguinal hernia that is partially reducible during the examination. There was no attempt to orchiopexy before three years, likely due to family social issues. The procedure was surgical laparoscopic exploration under general anesthesia; the right and left spermatic cords were passed through the right internal inguinal ring with both testes on the right side (Figs. 2,3). A laparoscopic procedure was converted to open and open herniotomy with lengthening of the spermatic cord was performed. The right testis was fixed to the left hemiscrotum through the trans-septal approach. The left testis was fixed to the right hemiscrotum via sub-dartos fixation. Postoperatively, the patient tolerated the surgical procedure without any complications. The patient was followed up in the clinic for six months postoperative. Upon examination, both testes were palpable and of good size in the scrotum.

We did a literature review of 5 patients diagnosed with Transverse Testicular Ectopia (TTE), summarizing the presenting symptoms, surgical methods, and outcome (Table 1).

**Table 1:** Descriptive summary of cases We summarize the cases with demographics, presenting symptoms, surgical procedures, and postoperative outcomes

Article	Author	Patient Age	Symptoms	Type of Surgery	Post-Op complications
Transverse testicular ectopia: two case reports and literature review.	Our Case	Case 1: 2 years Case 2: 3 years	Case 1: - Impalpable left testis - Inguinal area slightly enlarged Case 2: - Left impalpable undescended testis - Right inguinal hernia which is partially reducible during examination	Case 1: Herniotomy Case 2: Laparoscopic exploration and open herniotomy	Case 1: None Case 2: None
Transvers testicular ectopia: A case report and literature review	Abdullayev T et al., 2019	8 months	1- Bilateral undescended testes 2- Left undescended testis in inguinal canal 2- Unpalpable Right testis	Diagnostic laparoscopy and bilateral orchiopexy	NR
Transverse Testicular Ectopia	Muraveji Q et al., 2020	16 months	1- Large right hemiscrotum 2- Unpalpable left testes	Orchidopexy	None
Transverse testicular ectopia associated with persistent Mullerian duct syndrome in infertile male: two case reports and literature review	Yang C et al., 2021	Case 1: 28 years Case 2: 28 years	Case 1: Bilateral impalpable testes Case 2: - 2 cm mass in right inguinal region - Bilateral impalpable testes and empty scrotum	Case 1: Bilateral testicular descending orchiopexy with transperitoneal laparoscopy Case 2: laparoscopic herniorrhaphy	Case 1: None Case 2: None
Transverse testicular ectopia associated with persistent Müllerian duct syndrome treated by transseptal orchiopexy	Chung HS et al., 2018	2 years	1- Palpatable mass in left inguinal area 2- Bilateral impalpable testes	Transseptal Orchiopexy	NR
“Transverse testicular ectopia with inguinal hernia in an adult—a case report”	Pitchumani S et al., 2021	33 years	1- Non-reducible right inguinoscrotal swelling 2- Unpalpable right testes	Hernioplasty	NR

## 3 Discussion

Transverse Testicular Ectopia (TTE) is a congenital abnormality that is characterized by descending the testis through the same inguinal canal toward the hemiscrotum [1] [2]. Furthermore, it usually occurs in patients with undescended testes or operated for inguinal hernia [3]. Transverse Testicular Ectopia (TTE) is classified into three types according to the patient's clinical presentation [3]. The most common type is Type 1, associated with inguinal hernias, with an occurrence rate of 40–50 % of cases [3]. Type 2 is associated with persistent Mullerian duct syndrome (PMDS) and occurs in 30 % of cases [3]. Type 3 is associated with hypospadias, scrotal anomalies, fused vas deferens, seminal vesicle cysts, and testicular microlithiasis, which occurs in 20 % of cases [3]. The embryological development is associated with several factors; Gupt et al. explained that the Wolffian duct's early fusion and adherence can result in one testis's descent, causing the second testes to follow [5]. Paltii et al. showed that defects in the implantation of the testicular gubernaculum or inguinal ring obstruction can prevent the testicles from descending to the ipsilateral side [5]. Josso et al. showed abnormal adherence of the testes to adjacent structures can contribute to the development of Transverse Testicular Ectopia (TTE) [5].

The exact pathogenesis is not well understood [3]. However, studies suggest the involvement of a persistent Mullerian duct, which prevents the testicles from descending usually or pushes the testicles to the same hemiscrotum, resulting in the development of Transverse Testicular Ectopia (TTE) [3]. The clinical presentation can vary depending on the associated anomalies and age at diagnosis [1]. Transverse Testicular Ectopia (TTE) is usually asymptomatic and discovered incidentally during surgery or via imaging [1] [3]. The hernia presents as a palpable mass in the groin region [1]. The diagnosis requires clinical evaluation, image modalities, and surgical investigation. Physical examination can reveal non-palpable testes in the scrotum and contralateral reducible or irreducible hernia [3]. Image modalities such as ultrasonography, magnetic resonance imaging (MRI), and laparoscopy can assess any abnormal position in the testes [1] [3]. Surgical exploration is made to confirm the diagnosis and assess potential damage to associated structures or abnormalities.

Surgical outcomes depend on several factors, including the location and testicular degeneration, which can affect the choice of operation [2]. Different surgical interventions, including orchiopexy, hernia repair, and reduction of the testis, are performed in other approaches, either an inguinal approach, laparoscopy, laparoscopic-assisted inguinal approach, or laparotomy [3]. Orchiopexy is commonly performed for patients with Transverse Testicular Ectopia (TTE), and it can be done through different approaches, including trans-septal or trans-peritoneal procedures [3]. *trans*-Septal orchiopexy (Ombredanne's technique) is the preferred modality and is usually done alongside the repair of the inguinal hernia and congenital anomalies [3]. The procedure is performed by separating the anomalous testis from the cord structures and the hernia sac on the same side and transferring them to the hemiscrotum on the opposite side.

In contrast, the trans-peritoneal approach is made by placing the anomalous testis on the extra-peritoneal area by crossing the root of the penis and fixing it into the other side of the hemiscrotum [3]. The goal is to restore the testes and prevent further complications [2]. As with any procedure, complications can occur in such cases. This includes testicular atrophy, damage or ligation of vas deferense, infection, and testicular ascent [3]. Therefore, neglecting or delaying the treatment course can increase the risk of infertility in 6 % of cases [7]. Walsh et al. showed that cryptorchid cases can increase the risk of developing testicular cancer nearly six times higher if left untreated. Wood et al. reported that the risk of malignant transformation in undescended testes decreases when orchiopexy is done before the age of 12 years [8]. In our case, both patients tolerated the procedure, didn't develop any postoperative complications, and were scheduled for follow-up.

## 4 Conclusion

Transverse Testicular Ectopia (TTE) is an extremely rare anomaly documented in the literature. This case highlights the importance of early diagnosis and surgical treatment in young patients with impalpable testis coexisting with inguinal hernia to prevent long-term complications.

## Patient consent

Consent to publish the case series was not obtained. This report does not contain any personal information that could lead to the identification of the patient.

## Methods

The work has been reported in line with the SCARE criteria.

Agha RA, Franchi T, Sohrab C, Mathew G, Kirwan A, Thomas A, et al. The SCARE 2020 guideline: updating consensus Surgical Case Report (SCARE) guidelines. International Journal of Surgery. 2020; 84(1):226–30.

## Ethical approval

This is a case report and according DHHS is a medical/educational activity that does not meet the DHHS definition of “research”.

## Funding sources

There were no funding sources.

## Author contribution

A. Gamal Sayed, F. Joueidi, H, Sayed participated in writing the background with significance, case presentations, discussion, conclusion, read and approved the final manuscript.

A. Abokrecha, L. Alzahrani, F. Joueidi helped draft the manuscript, read, and agree on the final manuscript, and formatted the manuscript according to the journal's guidelines.

A. Abokrecha, L. Alzahrani, supervising the overall research, preparing the manuscript reading, and approving the final manuscript.

## Guarantor

Ahmed Abokrecha accepts full responsibility for the work and/or the conducted of the study, had access to the data and controlled the decision to publish.

## Conflict of interest statement

The authors declare that they have no known competing financial interests or personal relationships that could have appeared to influence the work reported in this paper.
